# Hexa­kis­(dimethyl­formamide-κ*O*)manganese(II) μ-oxido-bis­[trichlorido­ferrate(III)]

**DOI:** 10.1107/S1600536811041523

**Published:** 2011-10-22

**Authors:** Eduard N. Chygorin, Svitlana R. Petrusenko, Volodymyr N. Kokozay, Yuri O. Smal, Irina V. Omelchenko, Oleg V. Shishkin

**Affiliations:** a Department of Inorganic Chemistry, Taras Shevchenko National University of Kyiv, 64 Volodymyrs’ka St., Kyiv 01601, Ukraine; b STC "Institute for Single Crystals" National Academy of Sciences of Ukraine, 60 Lenina Avenue, Kharkiv 61001, Ukraine

## Abstract

The title compound, [Mn(C_3_H_7_NO)_6_][Fe_2_Cl_6_O], was obtained unintentionally as a product of an attempted synthesis of heterometallic complexes with Schiff base ligands using manganese powder and FeCl_3_·6H_2_O as starting materials. In the [Fe_2_OCl_6_]^2−^ anion, the O atom and the Fe atom occupy positions with site symmetry 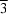 and 3, respectively, resulting in a linear Fe—O—Fe angle and a staggered conformation. The octa­hedrally surrounded cation (site symmetry 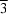) and the [Fe_2_Cl_6_O]^2−^ anion are alternately stacked along [001].

## Related literature

For structures including [Mn(dmf)_6_]^2+^ cations, see: Khutornoi *et al.* (2002 ▶). For stuctures including [Fe(dmf)_6_]^2+^, see: Albanati *et al.* (2007 ▶); Baumgartner (1986 ▶); Li *et al.* (2007**a* ▶,b* ▶); Lode & Krautscheid (2000 ▶); Müller *et al.* (1989**a* ▶,b* ▶); Qiutian *et al.* (1983 ▶); Silva *et al.* (2008 ▶); Young *et al.* (1989 ▶). For the isostructural complex [Mg(dmf)_6_][Fe_2_OCl_6_], see: Juang *et al.* (1984 ▶). For bond-valence-sum calculations, see: Brown & Altermatt (1985 ▶). For related direct syntheses, see: Garnovskii *et al.* (1999 ▶).
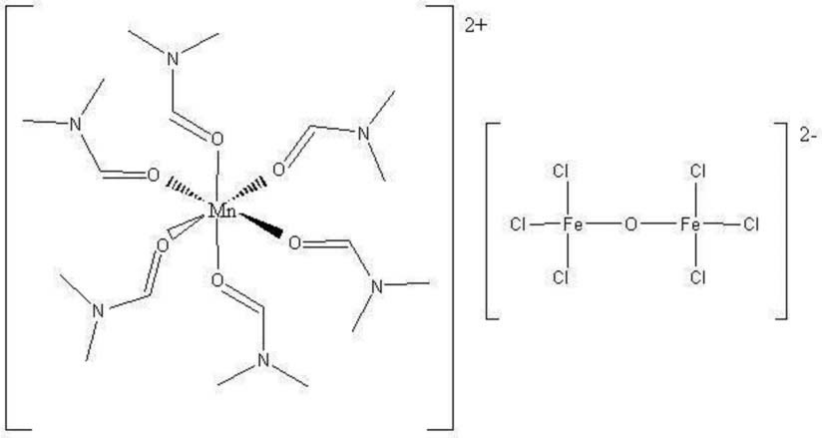


## Experimental

### 

#### Crystal data

[Mn(C_3_H_7_NO)_6_][Fe_2_Cl_6_O]
*M* *_r_* = 833.92
Trigonal, 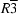

*a* = 14.0171 (8) Å
*c* = 15.3966 (14) Å
*V* = 2619.8 (3) Å^3^
*Z* = 3
Mo *K*α radiation
μ = 1.68 mm^−1^
*T* = 173 K
0.60 × 0.40 × 0.40 mm

#### Data collection

Oxford Diffraction Xcalibur/Sapphire3 diffractometer
Absorption correction: multi-scan (*CrysAlis RED*; Oxford Diffraction, 2010 ▶) *T*_min_ = 0.557, *T*_max_ = 1.000
16066 measured reflections
1624 independent reflections
1323 reflections with *I* > 2σ(*I*)
*R*_int_ = 0.067

#### Refinement

*R*[*F* ^2^ > 2σ(*F* ^2^)] = 0.039
*wR*(*F* ^2^) = 0.092
*S* = 0.98
1624 reflections
68 parameters
H atoms treated by a mixture of independent and constrained refinement
Δρ_max_ = 1.05 e Å^−3^
Δρ_min_ = −0.37 e Å^−3^

### 

Data collection: *CrysAlis CCD* (Oxford Diffraction, 2010 ▶); cell refinement: *CrysAlis RED* (Oxford Diffraction, 2010 ▶); data reduction: *CrysAlis RED*; program(s) used to solve structure: *SHELXTL* (Sheldrick, 2008 ▶); program(s) used to refine structure: *SHELXTL*; molecular graphics: *XP* in *SHELXTL*; software used to prepare material for publication: *publCIF* (Westrip, 2010 ▶).

## Supplementary Material

Crystal structure: contains datablock(s) I, global. DOI: 10.1107/S1600536811041523/wm2531sup1.cif

Structure factors: contains datablock(s) I. DOI: 10.1107/S1600536811041523/wm2531Isup2.hkl

Additional supplementary materials:  crystallographic information; 3D view; checkCIF report

## Figures and Tables

**Table 1:** Selected bond lengths (Å)

Mn1—O1	2.1736 (15)
Fe1—O1*S*	1.7758 (5)
Fe1—Cl1	2.2330 (6)

**Fig. 1.**
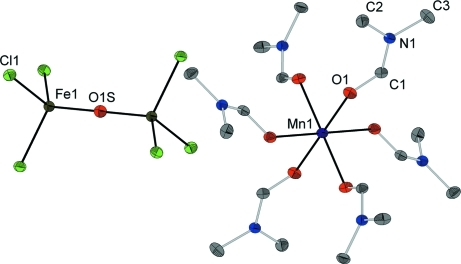
Parts of the structure of (I), with atom labels and 50% probability displacement ellipsoids. Hydrogen atoms were omitted for clarity.

**Fig. 2.**
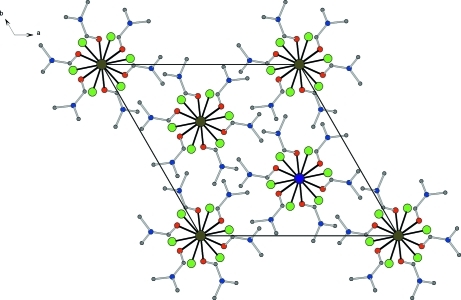
The crystal packing of (I) showing a staggerded conformation of the [Fe2OCl6]2- anion. Colour code as in Fig. 1; hydrogen atoms were omitted for clarity.

**Fig. 3.**
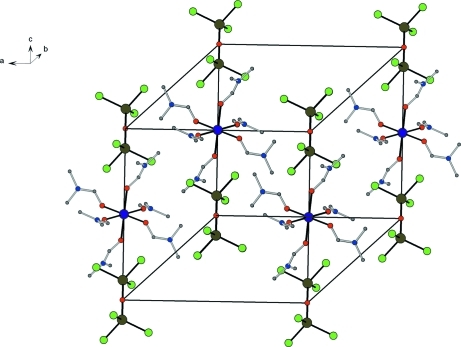
The crystal packing of (I) showing the linear arrangement of Fe–O–Fe mojeties and Mn atoms. Colour code as in Fig. 1; hydrogen atoms were omitted for clarity.

## supplementary crystallographic information

### Comment

Continuing our research on direct synthesis of bimetallic complexes with Schiff base ligands, *i.e.* one-pot synthesis with the use of metal powders or their oxides as starting materials (Garnovskii *et al.*, 1999), we present here a new Mn^II^/Fe^III^ complex, which was obtained as a by-product during the investigation of the system: Mn^0^ – FeCl_3_.6H_2_O – salicylic aldehyde – glycine – Et_3_N – dmf (dimethylformamide).

The crystal structure of the title complex, (I), is shown in Fig. 1. Six dmf molecules coordinate to the Mn^2+^ ion forming the almost regular octahedral complex cation [Mn(dmf)_6_]^2+^ [site symmetry 3; Mn1—O1 distances of 2.1736 (15) Å and O1–Mn1–O1 bond angles of 87.49 (6), 92.51 (6) and 180°]. Since the neighbouring elements Mn and Fe are difficult to distinguish by X-ray methods, a comparison of Mn^II^—O and Fe^II^—O bond lengths can be useful in the assignment of the correct metal to this polyhedron. The observed metal–O_dmf_ distances are in good agreement with those in [Mn(dmf)_6_][Mo_6_Br_8_(NCS)_6_] [2.152Å (Khutornoi *et al.*, 2002)]. An additional argument for Mn in the cation as the correct metal, but not its Fe analogue, is the analysis of crystallographic data for 10 structures containing [Fe(dmf)_6_]^2+^ (Albanati *et al.*, 2007; Baumgartner, 1986; Li *et al.*, 2007**a*,b*; Lode & Krautscheid, 2000; Müller *et al.*, 1989**a*,b*; Qiutian *et al.*, 1983; Silva *et al.*, 2008; Young *et al.*, 1989) where Fe—O bond lengths in the range of 1.99 – 2.16 Å with an average value of 2.11 Å are found. Moreover, no Fe^II^ complexes with a regular octahedral dmf environment have been found.

The [Fe_2_OCl_6_]^2-^ anion possesses *D*_3_*_d_* geometry with Fe and O atoms in special positions (3 and 3, respectively) that force the Fe—O—Fe angle to be linear and the anion to adopt a staggered conformation (Fig. 2). The Fe^3+^ atom has a distorted tetrahedral coordination with an Fe1–O1S distances of 1.7758 (5) Å, an Fe1–Cl1 distance of 2.2330 (6) Å, and bond angles of 106.348 (19) and 112. 438 (17)° for Cl1–Fe1–Cl1 and Cl1–Fe1–O1S, respectively. The oxidation states (+II) and (+III) for hexa-coordinate Mn and four-coordinate Fe atoms are supported by bond-valence sum calculations [1.914 for Mn^2+^ and 3.099 for Fe^3+^ (Brown & Altermatt, 1985)].

The [Mn(dmf)_6_]^2+^ cations and [Fe_2_OCl_6_]^2-^ anions are arranged in such a way that the cations and anions are arranged alternatingly along the [001] direction (Fig. 3).

The described complex (I) can be supposed as isostructural to [Mg(dmf)_6_][Fe_2_OCl_6_] (Juang *et al.*, 1984) but interestingly the volume of the unit cell in case of the manganese complex is *ca* 200 Å^3^ less than that of the magnesium complex.

### Experimental

Salicylic aldehyde (0.31 g, 2.5 mmol), glycine (0.19 g, 2.5 mmol) and triethylamine (0.35 mmol, 2.5 mmol) were dissolved in dimethylformamide (dmf; 25 ml) in this order, and stirred at 323 – 333 K (10 min). Then, manganese powder (0.14 g, 2.5 mmol) and FeCl_3_.6H_2_O (0.68 g, 2.5 mmol) were added to the hot yellow solution with stirring for 3 h, until total dissolution of manganese was observed. The resulting solution was filtered and subsequently pale pink crystals suitable for X-ray crystallography were separated after eight days at successive addition of a Pr*^i^*OH. Yield: 0.23 g, 21.5% (per iron). Elemental analysis for C_18_H_42_MnFe_2_N_6_O_7_Cl_6_ (Mr= 833.92). Calcd: C, 25.93; N, 10.08; H, 5.08; Fe, 13.39; Mn, 6.59. Found: C, 26.1; N, 10.0; H, 5.0; Fe, 13.3; Mn, 6.3. IR(KBr, cm^-1^): 3344(br), 2924(*m*), 1629(*m*), 1572(w), 1557(w), 1535(w), 1521(w), 1470(w), 1412(br), 1312(w), 1580(w), 1174(w), 1154(w), 1041(w), 952(w), 851(w), 689(*s*), 482(w), 443(w). The compound is sparingly soluble in dimethylsulfoxide (dmso), dmf, and H_2_O. In the IR spectrum of (I), the band corresponding to ν(CO) in dmf is shifted to the region of longer wavelenghts (1629 cm^-1^) relative to this band in the spectrum of noncoordinating dmf (1675 cm^-1^).

### Refinement

The carbonyl H atom was found from a difference Fourier map and was refined freely. Methyl H atoms were allowed to ride on their attached atoms with C—H = 0.98 (1)Å and *U*_iso_(H)= 1.5Ueq(C). The highest remaining difference Fourier peak is located 0.87 Å from atom O1S.

### Crystal data

**Table:**

[Mn(C_3_H_7_NO)_6_][Fe_2_Cl_6_O]	*D*_x_ = 1.586 Mg m^−^^3^
*M**_r_* = 833.92	Mo *K*α radiation, λ = 0.71073 Å
Trigonal, *R*3	Cell parameters from 2653 reflections
Hall symbol: -R 3	θ = 2.9–32.5°
*a* = 14.0171 (8) Å	µ = 1.68 mm^−^^1^
*c* = 15.3966 (14) Å	*T* = 173 K
*V* = 2619.8 (3) Å^3^	Block, red
*Z* = 3	0.60 × 0.40 × 0.40 mm
*F*(000) = 1281	

### Data collection

**Table:**

Oxford Diffraction Xcalibur/Sapphire3 diffractometer	1624 independent reflections
Radiation source: Enhance (Mo) X-ray Source	1323 reflections with *I* > 2σ(*I*)
graphite	*R*_int_ = 0.067
Detector resolution: 16.1827 pixels mm^-1^	θ_max_ = 30.0°, θ_min_ = 2.9°
ω scans	*h* = −19→19
Absorption correction: multi-scan (*CrysAlis RED*; Oxford Diffraction, 2010)	*k* = −19→15
*T*_min_ = 0.557, *T*_max_ = 1.000	*l* = −20→18
16066 measured reflections	

### Refinement

**Table:**

Refinement on *F*^2^	Primary atom site location: structure-invariant direct methods
Least-squares matrix: full	Secondary atom site location: difference Fourier map
*R*[*F*^2^ > 2σ(*F*^2^)] = 0.039	Hydrogen site location: difference Fourier map
*wR*(*F*^2^) = 0.092	H atoms treated by a mixture of independent and constrained refinement
*S* = 0.98	*w* = 1/[σ^2^(*F*_o_^2^) + (0.0428*P*)^2^] where *P* = (*F*_o_^2^ + 2*F*_c_^2^)/3
1624 reflections	(Δ/σ)_max_ < 0.001
68 parameters	Δρ_max_ = 1.05 e Å^−^^3^
0 restraints	Δρ_min_ = −0.37 e Å^−^^3^
0 constraints	

### Special details

**Table:**

Geometry. All e.s.d.'s (except the e.s.d. in the dihedral angle between two l.s. planes) are estimated using the full covariance matrix. The cell e.s.d.'s are taken into account individually in the estimation of e.s.d.'s in distances, angles and torsion angles; correlations between e.s.d.'s in cell parameters are only used when they are defined by crystal symmetry. An approximate (isotropic) treatment of cell e.s.d.'s is used for estimating e.s.d.'s involving l.s. planes.
Refinement. Refinement of *F*^2^ against ALL reflections. The weighted *R*-factor *wR* and goodness of fit *S* are based on *F*^2^, conventional *R*-factors *R* are based on *F*, with *F* set to zero for negative *F*^2^. The threshold expression of *F*^2^ > σ(*F*^2^) is used only for calculating *R*-factors(gt) *etc*. and is not relevant to the choice of reflections for refinement. *R*-factors based on *F*^2^ are statistically about twice as large as those based on *F*, and *R*- factors based on ALL data will be even larger.

### Fractional atomic coordinates and isotropic or equivalent isotropic displacement parameters (Å^2^)

**Table:**

	*x*	*y*	*z*	*U*_iso_*/*U*_eq_	
Mn1	0.0000	0.0000	0.5000	0.02020 (19)	
N1	0.23962 (14)	−0.02345 (15)	0.65588 (11)	0.0223 (4)	
Fe1	0.0000	0.0000	0.88467 (3)	0.02113 (16)	
O1	0.08798 (13)	−0.06054 (12)	0.57785 (10)	0.0262 (4)	
Cl1	0.03397 (5)	−0.12729 (4)	0.82931 (3)	0.02609 (16)	
C1	0.15838 (18)	−0.00668 (18)	0.63362 (15)	0.0232 (4)	
H1	0.1622 (18)	0.0567 (19)	0.6623 (13)	0.015 (6)*	
O1S	0.0000	0.0000	1.0000	0.0282 (8)	
C2	0.2521 (2)	−0.1115 (2)	0.61800 (16)	0.0318 (5)	
H2A	0.1939	−0.1512	0.5750	0.048*	
H2B	0.2467	−0.1626	0.6638	0.048*	
H2C	0.3242	−0.0802	0.5897	0.048*	
C3	0.3184 (2)	0.0425 (2)	0.72297 (17)	0.0347 (6)	
H3A	0.3143	−0.0055	0.7709	0.052*	
H3B	0.3010	0.0977	0.7446	0.052*	
H3C	0.3929	0.0795	0.6985	0.052*	

### Atomic displacement parameters (Å^2^)

**Table:**

	*U*^11^	*U*^22^	*U*^33^	*U*^12^	*U*^13^	*U*^23^
Mn1	0.0176 (2)	0.0176 (2)	0.0254 (4)	0.00880 (12)	0.000	0.000
N1	0.0200 (9)	0.0211 (9)	0.0259 (9)	0.0104 (7)	−0.0009 (7)	0.0000 (7)
Fe1	0.02076 (19)	0.02076 (19)	0.0219 (3)	0.01038 (10)	0.000	0.000
O1	0.0238 (8)	0.0209 (8)	0.0340 (9)	0.0112 (7)	−0.0040 (6)	0.0015 (6)
Cl1	0.0271 (3)	0.0233 (3)	0.0303 (3)	0.0145 (2)	0.0021 (2)	0.00083 (19)
C1	0.0209 (11)	0.0185 (10)	0.0296 (11)	0.0093 (9)	0.0026 (8)	0.0041 (8)
O1S	0.0274 (13)	0.0274 (13)	0.030 (2)	0.0137 (6)	0.000	0.000
C2	0.0373 (14)	0.0370 (13)	0.0331 (13)	0.0278 (12)	−0.0034 (10)	−0.0044 (10)
C3	0.0339 (13)	0.0269 (13)	0.0446 (14)	0.0162 (11)	−0.0151 (11)	−0.0065 (10)

### Geometric parameters (Å, °)

**Table:**

Mn1—O1^i^	2.1736 (15)	Fe1—Cl1	2.2330 (6)
Mn1—O1^ii^	2.1736 (15)	Fe1—Cl1^v^	2.2330 (6)
Mn1—O1	2.1736 (15)	O1—C1	1.239 (3)
Mn1—O1^iii^	2.1736 (15)	C1—H1	0.97 (2)
Mn1—O1^iv^	2.1736 (15)	O1S—Fe1^vi^	1.7758 (5)
Mn1—O1^v^	2.1736 (15)	C2—H2A	0.9800
N1—C1	1.318 (3)	C2—H2B	0.9800
N1—C2	1.453 (3)	C2—H2C	0.9800
N1—C3	1.456 (3)	C3—H3A	0.9800
Fe1—O1S	1.7758 (5)	C3—H3B	0.9800
Fe1—Cl1^ii^	2.2330 (6)	C3—H3C	0.9800
			
O1^i^—Mn1—O1^ii^	180.00 (6)	O1S—Fe1—Cl1^v^	112.438 (17)
O1^i^—Mn1—O1	87.49 (6)	Cl1^ii^—Fe1—Cl1^v^	106.348 (19)
O1^ii^—Mn1—O1	92.51 (6)	Cl1—Fe1—Cl1^v^	106.348 (19)
O1^i^—Mn1—O1^iii^	92.51 (6)	C1—O1—Mn1	125.35 (15)
O1^ii^—Mn1—O1^iii^	87.49 (6)	O1—C1—N1	124.6 (2)
O1—Mn1—O1^iii^	180.00 (7)	O1—C1—H1	122.4 (13)
O1^i^—Mn1—O1^iv^	92.51 (6)	N1—C1—H1	112.8 (13)
O1^ii^—Mn1—O1^iv^	87.49 (6)	Fe1^vi^—O1S—Fe1	180.0
O1—Mn1—O1^iv^	87.49 (6)	N1—C2—H2A	109.5
O1^iii^—Mn1—O1^iv^	92.51 (6)	N1—C2—H2B	109.5
O1^i^—Mn1—O1^v^	87.49 (6)	H2A—C2—H2B	109.5
O1^ii^—Mn1—O1^v^	92.51 (6)	N1—C2—H2C	109.5
O1—Mn1—O1^v^	92.51 (6)	H2A—C2—H2C	109.5
O1^iii^—Mn1—O1^v^	87.49 (6)	H2B—C2—H2C	109.5
O1^iv^—Mn1—O1^v^	180.00 (7)	N1—C3—H3A	109.5
C1—N1—C2	121.94 (18)	N1—C3—H3B	109.5
C1—N1—C3	121.34 (19)	H3A—C3—H3B	109.5
C2—N1—C3	116.65 (18)	N1—C3—H3C	109.5
O1S—Fe1—Cl1^ii^	112.438 (17)	H3A—C3—H3C	109.5
O1S—Fe1—Cl1	112.438 (17)	H3B—C3—H3C	109.5
Cl1^ii^—Fe1—Cl1	106.348 (19)		
			
O1^i^—Mn1—O1—C1	169.64 (18)	Mn1—O1—C1—N1	−152.23 (16)
O1^ii^—Mn1—O1—C1	−10.36 (18)	C2—N1—C1—O1	−2.2 (3)
O1^iv^—Mn1—O1—C1	77.01 (14)	C3—N1—C1—O1	−179.2 (2)
O1^v^—Mn1—O1—C1	−102.99 (14)		

Symmetry codes: (i) *y*, −*x*+*y*, −*z*+1; (ii) −*y*, *x*−*y*, *z*; (iii) −*x*, −*y*, −*z*+1; (iv) *x*−*y*, *x*, −*z*+1; (v) −*x*+*y*, −*x*, *z*; (vi) −*x*, −*y*, −*z*+2.

## References

[bb1] Albanati, A., Calderazzo, F., Marchetti, F., Mason, S. A., Melai, B., Pampaloni, G. & Rizatto, S. (2007). *Inorg. Chem. Commun.* **10**, 902–904.

[bb2] Baumgartner, O. (1986). *Z. Kristallogr.* **174**, 253–263.

[bb3] Brown, I. D. & Altermatt, D. (1985). *Acta Cryst.* B**41**, 244–247.

[bb4] Garnovskii, A. D., Kharisov, B. I., Skopenko, V. V., Blanco Jerez, L. M., Kokozay, V. N., Kuzharov, A. S., Garnovskii, D. A., Vassilyeva, O. Yu., Burlov, A. S. & Pavlenko, V. A. (1999). *Direct Synthesis of Coordination and Organometallic Compounds.* Amsterdam: Elsevier.

[bb5] Juang, L., Jiang, F. & Lu, J. (1984). *Huaxue Tongbao*, **3**, 14–19.

[bb6] Khutornoi, V. A., Naumov, N. G., Mironov, Yu. V., Oeckler, O., Simon, A. & Fedorov, V. E. (2002). *Russ. J. Coord. Chem.* **28**, 193–201.

[bb7] Li, Y., Zhang, Z.-X., Li, K.-C., Song, W.-D., Cui, X.-B. & Pan, L.-Y. (2007*a*). *J. Mol. Struct.* **843**, 102–106.

[bb8] Li, Y., Zhang, Z.-X., Li, K.-C., Xu, J.-Q., Song, W.-D. & Pan, L.-Y. (2007*b*). *J. Mol. Struct.* **833**, 8–12.

[bb9] Lode, C. & Krautscheid, H. (2000). *Z. Anorg. Allg. Chem.* **626**, 326–331.

[bb10] Müller, A., Bogge, H., Schimanski, U., Penk, M., Nieradzik, K., Dartmann, M., Krickemeyer, E., Schimanski, J., Romer, C., Romer, M., Dornfeld, H., Wienboker, U., Hellmann, W. & Zimmermann, M. (1989*a*). *Monatsh. Chem.* **120**, 367–391.

[bb11] Müller, A., Schaladerbeck, N. H., Krickemeyer, E., Bogge, H., Schmitz, K., Bill, E. & Trautwein, A. X. (1989*b*). *Z. Anorg. Allg. Chem.* **570**, 7–36.

[bb12] Oxford Diffraction (2010). *CrysAlis CCD* and *CrysAlis RED* Oxford Diffraction Ltd, Yarnton, England.

[bb13] Qiutian, L., Liangren, H., Beisheng, K. & Jiaxi, L. (1983). *Chin. J. Struct. Chem.* **2**, 225–229.

[bb14] Sheldrick, G. M. (2008). *Acta Cryst.* A**64**, 112–122. 10.1107/S0108767307043930 18156677

[bb15] Silva, R. M., Gwengo, C., Lindeman, S. V., Smith, M. D., Long, G. J., Grandjean, F. & Gardinier, J. R. (2008). *Inorg. Chem.* **47**, 7233–7242. 10.1021/ic8005794 18646840

[bb16] Westrip, S. P. (2010). *J. Appl. Cryst.* **43**, 920–925.

[bb17] Young, A. C. M., Walters, M. A. & Dewan, J. C. (1989). *Acta Cryst.* C**45**, 1733–1736.

